# Sarcopenia in idiopathic pulmonary fibrosis: an updated systematic review and meta-analysis

**DOI:** 10.3389/fmed.2025.1681237

**Published:** 2025-11-04

**Authors:** Jun Zeng, Jia Liu, Liqian He, Jianyu Zhu, Jie He, Jiaqing Jiang, Zhenglin Chen, Xinrui Zhu, Jia He, Jingyue Yang, Yifan Yuan

**Affiliations:** ^1^School of Clinical Medicine, Chengdu Medical College, Chengdu, Sichuan, China; ^2^Department of Pulmonary and Critical Care Medicine, The First Affiliated Hospital of Chengdu Medical College, Chengdu, Sichuan, China; ^3^Key Laboratory of Geriatric Respiratory Diseases of Sichuan Higher Education Institutes, Chengdu, Sichuan, China; ^4^Department of General Practice, The First Affiliated Hospital of Chengdu Medical College, Chengdu, Sichuan, China

**Keywords:** IPF, meta-analysis, prevalence, pulmonary function, sarcopenia

## Abstract

**Background:**

Sarcopenia has frequently been identified as a comorbid condition in numerous diseases, ultimately influencing patient outcomes. Nonetheless, within the context of idiopathic pulmonary fibrosis (IPF), it has received relatively insufficient clinical focus. This systematic review and meta-analysis were conducted to estimate the prevalence of sarcopenia among individuals diagnosed with IPF and to investigate the links between sarcopenia and both pulmonary function and prognosis in this population.

**Methods:**

Comprehensive literature searches were carried out in Excerpta Medica Database (Embase), PubMed, Web of Science, China National Knowledge Infrastructure (CNKI), and Cochrane Library databases up to 7 July 2025, utilizing relevant MeSH terms. Quality evaluation was implemented through the Joanna Briggs Institute (JBI) tool and the Newcastle-Ottawa Scale. To account for heterogeneity across studies, a random-effects model was implemented. Statistical heterogeneity was examined utilizing I^2^ statistics. The overall prevalence of sarcopenia was calculated by pooling data to generate percentages accompanied by 95% confidence intervals (CI). For continuous variables sharing identical units, weighted mean differences were adopted as effect size metrics.

**Results:**

In total, 15 studies satisfied the eligibility requirements for inclusion. Findings indicated that sarcopenia was present in 25% of IPF cases. Participants were stratified into subgroups based on factors such as ethnicity, age, and diagnostic standards for sarcopenia to facilitate subgroup meta-analyses. A consistently elevated prevalence of sarcopenia was observed across all subgroup classifications. Furthermore, most parameters used to assess sarcopenia were found to be decreased in IPF individuals, and those with sarcopenia demonstrated impaired pulmonary function, reduced exercise capacity, and an overall worse prognosis.

**Conclusion:**

Current evidence supports the notion that sarcopenia is highly prevalent in IPF populations and may be closely linked to pulmonary functional impairment. Therefore, early identification and therapeutic intervention of sarcopenia should be emphasized in individuals with IPF. Moreover, standardized sarcopenia diagnostic criteria and protocols are urgently needed to ensure accurate meta-analysis results and research conclusions. A prospective, multicenter prospective study with inclusion of sex-specific and comorbidity-adjusted analyses should be awaited in the future.

**Systematic review registration:**

https://www.crd.york.ac.uk/PROSPERO/view/CRD420251089889, identifier CRD420251089889.

## 1 Introduction

IPF is recognized as an interstitial fibrotic disorder of unknown origin, marked by rapid clinical deterioration and unfavorable prognosis (1). The establishment of a definitive diagnosis necessitates the identification of hallmark features via high-resolution computed tomography and/or histopathological evidence obtained through lung biopsy, while simultaneously ruling out alternative known etiologies (2). Among individuals presenting with terminal respiratory insufficiency attributable to IPF, profound dyspnea, markedly diminished exercise capacity, and a decline in health-related quality of life (HRQOL) are commonly observed. This condition is further distinguished by its elevated rates of both morbidity and mortality (3).

IPF patients are frequently affected by disease-associated malnutrition, which is defined by the simultaneous presence of subacute or chronic inflammation and a persistent negative energy balance. This nutritional imbalance typically manifests through reduced BMI, decreased muscular capacity and power, alongside diminished phase angle (PhA) measurements (4, 5). Currently, several mechanisms have been implicated in the progressive decline of nutritional status and alterations in body composition, encompassing aging; vitamin D deficiency linked to smoking habits; gastrointestinal disturbances; involuntary weight loss; acute exacerbation episodes; increased load on respiratory musculature; and appetite-suppressing effects of antifibrotic agents such as nintedanib/pirfenidone (6–8). Additional contributing factors encompass reduced levels of physical activity stemming from the general clinical condition; systemic inflammatory responses; oxidative stress (OS); hypercatabolic states; inadequate protein consumption; reduced forced vital capacity (FVC); and comorbid diabetes (5–7, 9).

Alterations in body composition are primarily indicated by reductions in skeletal muscle mass, dynapenia, and sarcopenia. Empirical evidence has demonstrated that declines in body weight and skeletal muscle mass are strongly connected to reduced FVC percentage predicted (FVC%), impaired diffusing capacity of the lung for carbon monoxide (DLCO), and unfavorable clinical outcomes (10–12). Dynapenia, defined by diminished muscle strength, has been identified not only as a contributor to impaired muscular function (13) but also as a robust predictor of adverse outcomes, including extended hospitalization, exacerbation of functional limitations, decline in HRQOL, and increased mortality risk (14, 15). In patients with interstitial lung disease (ILD), decreased handgrip strength (HGS) has been shown to exhibit a significant inverse correlation with reduced FVC and the severity of dyspnea (16).

Sarcopenia refers to a systematic and ongoing condition affecting skeletal muscle, marked by decreased muscle strength and mass (17). Within the context of respiratory diseases, the presence of sarcopenia has been demonstrated to substantially elevate the risk of falls, fractures, and hospital admissions, while also contributing to diminished capacity for daily activities and unfavorable clinical outcomes (18–21). A meta-analysis conducted by Li (22), encompassing publications from 2019 to 2022, reported a sarcopenia prevalence of 26% among IPF patients and explored potential etiological factors underlying its development. The majority of included studies focused on Asian populations. Given that Caucasian individuals also constitute a high-risk demographic for IPF (23), the inclusion of ethnically diverse cohorts in future meta-analyses is warranted. Variability in sarcopenia prevalence among IPF patients may also be attributed to inconsistencies in diagnostic modalities employed to assess muscle mass. Moreover, an increasing number of investigations examining the coexistence of IPF and sarcopenia have emerged in recent years, often yielding divergent findings. In light of this, the present meta-analysis was undertaken to synthesize recent observational evidence, identify subgroup differences in sarcopenia prevalence among IPF patients, and examine its influence on pulmonary function and prognosis.

## 2 Materials and methods

The current systematic review and meta-analysis were executed per the Preferred Reporting Items for Systematic Reviews and Meta-Analyses Guidelines (PRISMA) 2020, and the protocol was registered with PROSPERO with the registration number CRD420251089889. The PRISMA 2020 checklist is presented in Supplementary Table 1.

### 2.1 Information sources

Relevant research literature was retrieved through comprehensive electronic searches of multiple databases, supplemented by manual screening of reference lists from eligible publications. The search timeframe spanned from the inception of each database to 1 August 2025, and included the following sources: PubMed, CNKI, Web of Science, Cochrane Library, and Embase.

### 2.2 Search strategy

This search sought to assess the prevalence of sarcopenia among individuals with IPF. The search strategy involved a combination of relevant descriptors and Boolean operators (AND/OR), including the following terms: (“Idiopathic Pulmonary Fibroses,” OR “Pulmonary Fibroses, Idiopathic,” OR “Idiopathic Fibrosing Alveolitis, Chronic Form,” OR “Fibrosing Alveolitis, Cryptogenic,” OR “Fibrocystic Pulmonary Dysplasia,” OR “Dysplasia, Fibrocystic Pulmonary,” OR “Fibrocystic Pulmonary Dysplasias,” OR “Pulmonary Dysplasia, Fibrocystic,” OR “Cryptogenic Fibrosing Alveolitis,” OR “Cryptogenic Fibrosing Alveolitides,” OR “Fibrosing Alveolitides, Cryptogenic,” OR “Pulmonary Fibrosis, Idiopathic,” OR “Familial Idiopathic Pulmonary Fibrosis,” OR “Idiopathic Pulmonary Fibrosis, Familial,” OR “Usual Interstitial Pneumonia,” OR “Usual Interstitial Pneumonias,” OR “Interstitial Pneumonitis, Usual,” OR “Pneumonitides, Usual Interstitial,” OR “Pneumonitis, Usual Interstitial,” OR “Usual Interstitial Pneumonitides,” OR “Usual Interstitial Pneumonitis”) AND (“sarcopenia,” OR “sarcopenic,” OR “muscle mass,” OR “muscle strength,” OR “hand strength,” OR “grip strength,” OR “muscle atrophy,” OR “muscle wasting”). Only publications written in either Chinese or English were considered for inclusion. The complete search syntax is depicted in Supplementary Table 2.

### 2.3 Selection criteria

Studies qualified for meta-analysis inclusion needed to meet these specifications: (1) original investigations focusing on sarcopenia in the context of IPF; (2) studies involving human participants; (3) assessment of sarcopenia; (4) application of valid and objective diagnostic approaches for IPF; (5) observational designs (cross-sectional or cohort studies) or clinical trials (randomized or non-randomized); and (6) reporting of sarcopenia prevalence either as a percentage or in a format permitting calculation from the presented data.

Exclusion criteria included the following: (1) publication types such as reviews, case reports, conference abstracts, commentaries, or editorials; (2) absence of a clearly defined sarcopenia diagnosis; and (3) evidently inaccurate or incomplete data.

### 2.4 Data extraction

Following the initial screening of titles and abstracts, the full texts of eligible studies were independently evaluated by two authors (Jie He and Jia Liu). Manual searches of reference lists from relevant publications were also conducted. The extracted data, independently cross-verified by both reviewers, included: first author, year of publication, country, study design, sample size, participant age, sex, Body Mass Index (BMI), smoking history, diagnostic criteria for sarcopenia, prevalence of sarcopenia, sarcopenia-related assessment parameters [skeletal muscle index (SMI), HGS, PhA, etc.], and clinical outcomes associated with sarcopenia, including pulmonary function indices, exercise capacity, mortality risk, and the proportion of participants receiving antifibrotic therapy. Disagreements underwent resolution through discussion between the two reviewers or through the involvement of a third author (Jiaqing Jiang).

### 2.5 Primary outcomes

Prevalence of sarcopenia in IPF patients.

### 2.6 Secondary outcomes

Measurements included HGS, SMI, timed up and go test (TUG), PhA, erector spinae muscle cross-sectional area (ESMCSA), and psoas muscle cross-sectional area (PMCSA) in patients with IPF. Additionally, differences were analyzed in the 6-min walk test (6MWT), DLCO percentage predicted (DLCO%), forced expiratory volume in 1 s (FEV1), FEV1 percentage predicted (FEV1%), FEV1/FVC ratio, FVC, and FVC% between IPF patients with sarcopenia and those without.

### 2.7 Literature quality assessment

The methodological quality of the included studies was assessed independently by a minimum of two authors (Jia Liu and Jie He) using a standardized assessment scale. Bias risk was assessed via the JBI critical appraisal checklist (Supplementary Table 3), which was specifically developed for studies presenting prevalence data. Based on the scoring criteria, studies were categorized as low-quality (grade C), medium-quality (grade B), or high-quality (grade A) when total scores fell within the ranges of 0–3, 4–6, and 7–9, respectively (24). For cross-sectional and cohort studies, the Newcastle-Ottawa Scale (NOS) was applied. NOS scores of 0–3, 4–6, and 7–9 corresponded to low, medium, and high quality, respectively (25).

### 2.8 Statistical analysis

This meta-analysis was conducted to evaluate three primary outcome measures: (1) the prevalence of sarcopenia among IPF patients; (2) assessment-related indicators of sarcopenia in individuals with IPF; and (3) differences in pulmonary function, use of anti-fibrotic agents, and prognosis between IPF patients with and without sarcopenia. To synthesize prevalence estimates, a single-proportion meta-analysis was applied, and subgroup analyses were performed based on sarcopenia diagnostic criteria, ethnicity, age group, and study design. For prevalence calculations, generalized linear mixed models were adopted, and either fixed-effects or random-effects models were selected following a logit transformation. Data processing was applied to the prevalence of sarcopenia data by meta packages and metaprop function in R software (version 4.1.3). In single-arm studies reporting sarcopenia-related indicators only, the standard error for each metric was computed using the metan package in STATA 11.0, and pooled analyses were subsequently carried out. When pulmonary function measures were reported separately for sarcopenic and non-sarcopenic patients, relevant continuous variables were extracted. Fixed-effects meta-analysis was utilized in cases of statistical homogeneity. Otherwise, a random-effects model was employed. Continuous outcomes measured on the same scale were described using weighted mean differences and standard deviations, whereas standardized mean differences were applied when outcomes were measured on different scales. For continuity variables, we used the current version of the Cochrane Review Manager software, RevMan 5.2. The pooled enumeration data were expressed as relative risk (RR) and 95% confidence interval (CI) by meta packages and the metabin function in R software (version 4.1.3). Heterogeneity assessment incorporated I^2^ and Cochran’s Q metrics, defining substantial heterogeneity at *P*-value ≤ 0.1 or I^2^ ≥ 50%. Heterogeneity source exploration involved descriptive plus stratified analyses. Sequential study elimination enabled sensitivity evaluation regarding heterogeneity impact. Publication bias underwent Begg’s and Egger’s test examination. Statistical significance threshold remained at two-tailed *P*-value < 0.05.

## 3 Results

### 3.1 Search results

**FIGURE 1:**
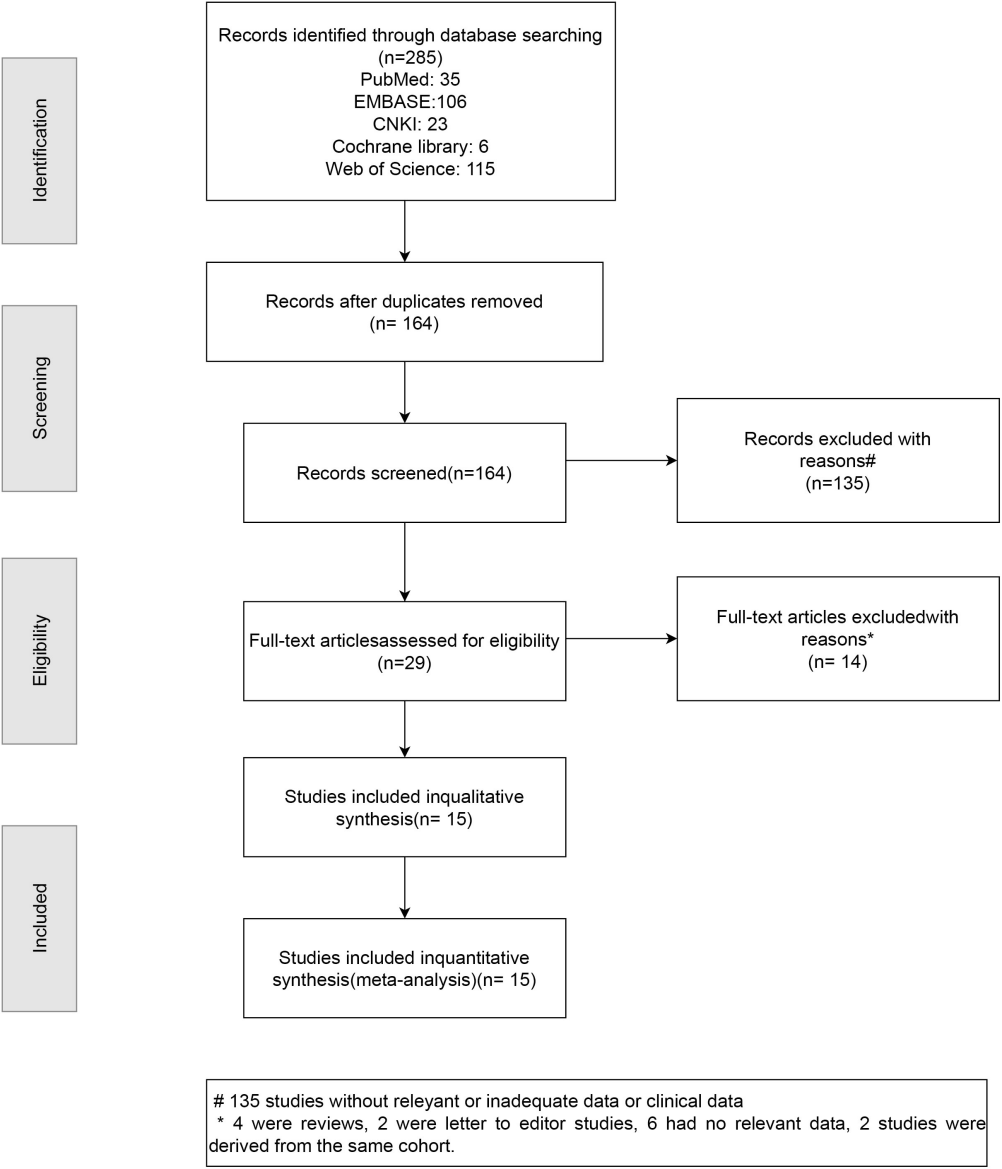
PRISMA flow diagram illustrating the selection process of studies included in the systematic review and meta-analysis.

**FIGURE 2:**
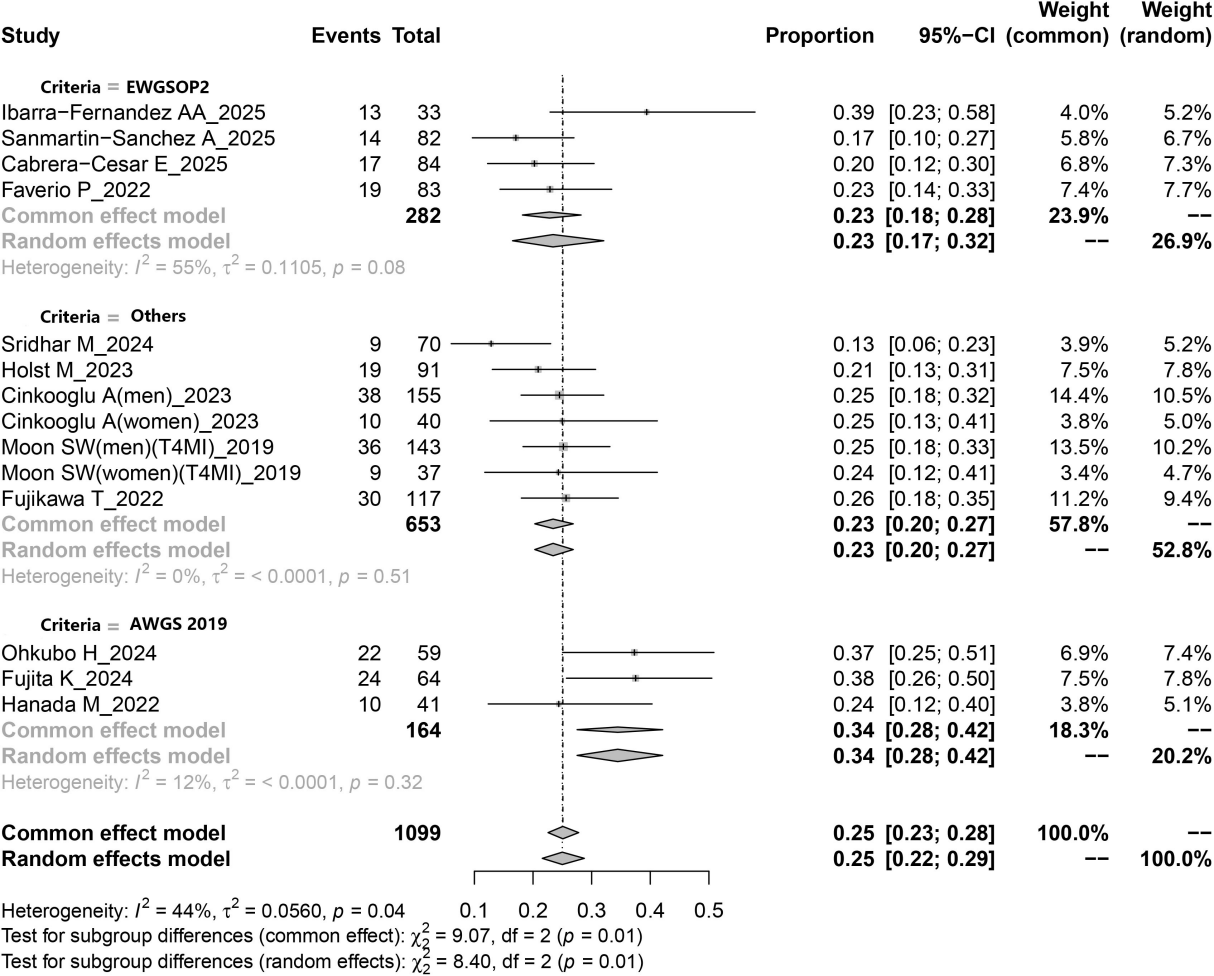
Forest plot depicting the pooled prevalence of sarcopenia among patients with IPF based on a random-effects model.

**FIGURE 3:**
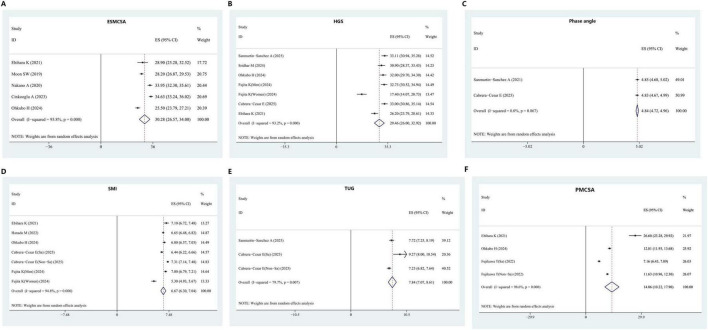
Assessment indicators of sarcopenia in IPF patients. **(A)** ESMCSA. **(B)** HGS. **(C)** Phase angle. **(D)** SMI. **(E)** TUG. **(F)** PMCSA. ESMCSA, erector spinae muscle cross-sectional area; HGS, handgrip strength; SMI, skeletal muscle index; TUG, timed up and go test; PMCSA, psoas muscle cross-sectional area.

**FIGURE 4:**
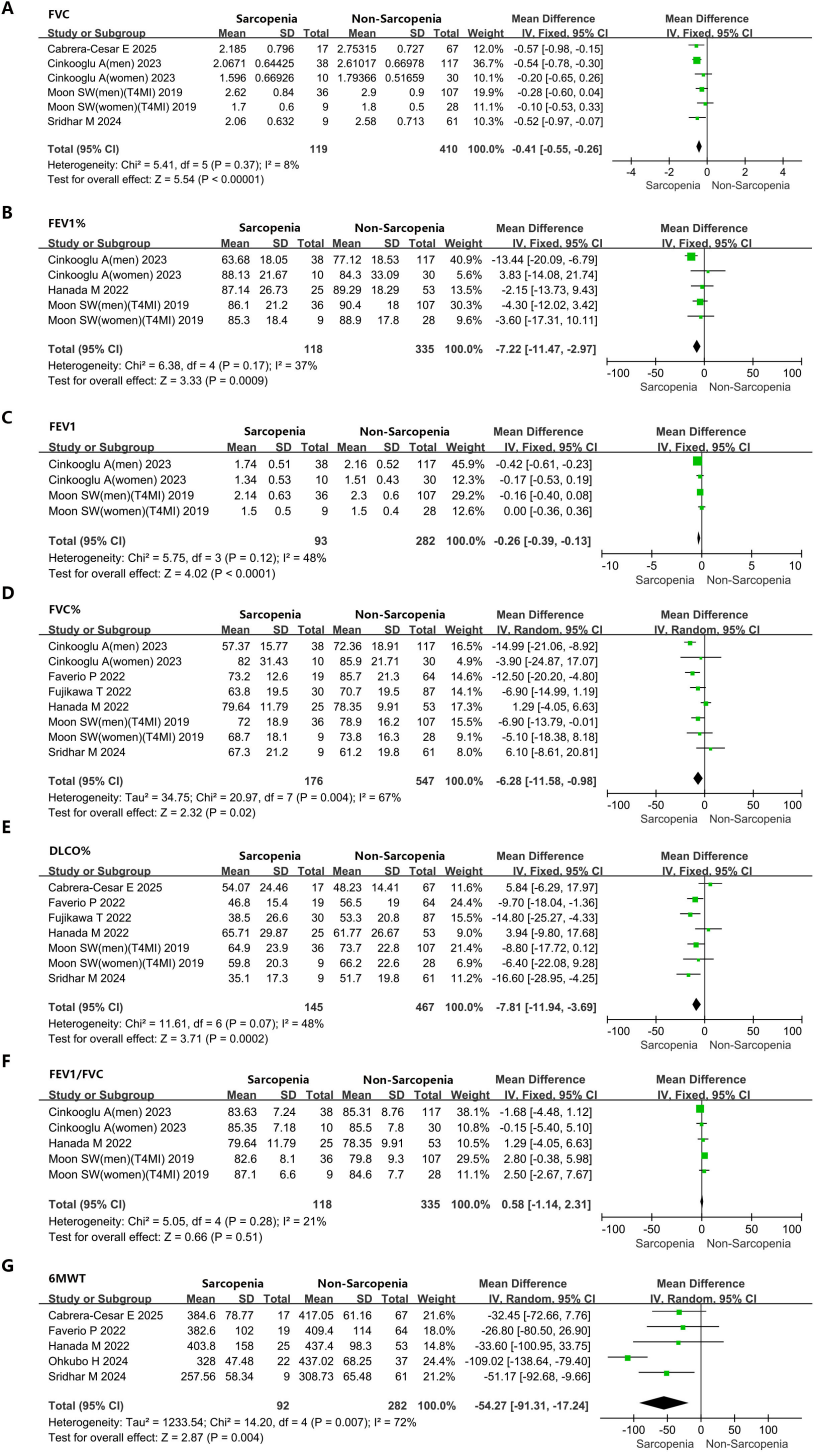
Forest plots showing the impact of sarcopenia on pulmonary function parameters and exercise capacity in IPF patients. **(A)** FVC. **(B)** FEV1%. **(C)** FEV1. **(D)** FVC%. **(E)** DLCO%. **(F)** FEV1/FVC. **(G)** 6MWT. FVC, forced vital capacity; FEV1%, forced expiratory volume in 1 second percentage predicted; FEV1, forced expiratory volume in 1 second; FVC%, forced vital capacity percentage predicted; DLCO%, diffusing capacity of the lung for carbon monoxide percentage predicted; FEV1/FVC, forced expiratory volume in 1 second/forced vital capacity; 6MWT, 6-min walk test.

The systematic review and meta-analysis selection methodology is depicted in Figure 1. Initially, 285 articles were discovered through searches in five primary databases. Following the removal of duplicates, 164 publications advanced to title and abstract review. Subsequently, 135 articles were excluded, leaving 29 for comprehensive assessment, ultimately yielding 15 qualified studies (12, 16, 26–38). The methodological assessment indicated “high” or “moderate” quality across selected studies. All 15 studies (12, 16, 26–38) reported data on sarcopenia prevalence (Figure 2). Among them, 10 studies (16, 27, 28, 31, 33–37) presented sarcopenia-related assessment parameters in IPF patients (Figure 3). Pulmonary function-related indicators were compared between sarcopenia-positive and sarcopenia-negative IPF patients in 8 studies (12, 16, 28, 29, 31–33, 35) (Figure 4). A sum of 5 studies (16, 28, 29, 33, 35) provided results for the 6-min walking distance (6MWD) between the two patient subgroups. Clinical outcomes in IPF patients concurrently diagnosed with sarcopenia were reported in 5 studies (16, 28, 31, 32, 38). Three studies described the number of IPF patients receiving anti-fibrotic therapy.

Table 1 presents the baseline characteristics of the included studies. Sample sizes ranged from 33 to 195 participants, with mean or median ages spanning from 63.89 to 74.4 years. Among the included studies, eight adopted prospective designs, while seven employed retrospective approaches. A variety of diagnostic criteria were applied, including the lowest quartile of T4-level muscle index (T4MI) (*n* = 1), erector spinae muscle area (ESMA) (*n* = 1), sarcopenia index (*n* = 1), strength, assistance with walking, rising from a chair, climbing stairs, and falls questionnaire (SARC-F) (*n* = 1), psoas muscle index (PMI) (*n* = 1), SMI (*n* = 1), pectoralis major cross-sectional area (PMCSA) (*n* = 2), the European Working Group on Sarcopenia in Older People 2019 criteria (EWGSOP2 2019) (*n* = 4), and the Asian Working Group for Sarcopenia 2019 criteria (AWGS 2019) (*n* = 3). Of these studies, seven were conducted in Asia, six in Europe, one in North America, and one in South America. Table 2 provides a summary of the sarcopenia diagnostic criteria utilized in the included studies.

**TABLE 1:** Characteristic of the included studies regarding the prevalence of sarcopenia in subjects with idiopathic pulmonary fibrosis.

References	Country	Year	Study design	Sample size	Age, (years)	Male, *n* (%)	BMI, kg/m^2^	Diagnostic criteria of sarcopenia	Quality/ degree
Nakano et al. (37)	Japan	2020	Retrospective CS	119	67.0 (61.0–71.0)	98 (82.4%)	23.4 (21.7–25.2)	(The lowest quartile of PMcas) Q4	6/B
Moon et al. (12)	Korea	2019	Retrospective CSS	180	69.1 (44–94)	143 (79.4%)	23.9 ± 3.2	(The lowest quartile of T4MI) Q4	8/A
Ebihara et al. (36)	Japan	2021	Retrospective CSS	27	76.1 ± 5.9	21 (78%)	24.0 ± 3.0	(The lowest quartile of SMI) Q4	7/A
Faverio et al. (29)	Italy	2022	Prospective CS	83	72.5 ± 6.5	67 (80.72%)	27.6 ± 4.0	EWGSOP2 2019	9/A
Hanada et al. (35)	Japan	2022	Prospective CCS	41	71 (67–77)	51 (65%)	24 (20–26)	AWGS 2019	5/B
Fujikawa et al. (32)	Japan	2022	Retrospective CS	117	74.4 ± 7.7	92 (78.63%)	22.9 ± 3.6	(The lowest quartile of PMI) Q4	6/B
Çinkooğlu et al. (31)	Turkey	2023	Retrospective CS	195	69.28 ± 7.52	155 (79.49%)	27.47 ± 4.07	(The lowest quartile of ESMA) Q4	6/B
Holst et al. (30)	Denmark	2023	Retrospective CS	91	70.5 (60–77)	65 (66.3%)	27.8 (24.4–30.4)	SARC-F	7/A
Fujita et al. (34)	Japan	2024	Prospective CSS	64	73.6 ± 7.9	57 (89.1%)	22.6 ± 3.3	AWGS 2019	6/B
Ohkubo et al. (33)	Japan	2024	Prospective CSS	59	73.3 ± 8.1	53 (89.83%)	22.6 ± 3.3	AWGS 2019	6/B
Sridhar et al. (16)	USA	2024	Prospective CS	70	70.4 ± 6.95	48 (68.5%)	29.4 ± 4.85	(The lowest quartile of PMcas) Q4	8/A
Cabrera-César et al. (28)	Spain	2025	Prospective CS	84	71 ± 7.22	71 (83.5%)	27.6 ± 3.47	EWGSOP2 2019	6/B
Ibarra-Fernández et al. (26)	Mexico	2025	Prospective CS	33	63.89 ± 12.02	37 (37.76%)	26.32 ± 4.94	EWGSOP2 2019	8/A
Sanmartín-Sánchez et al. (27)	Spain	2025	Prospective CSS	82	71.1 ± 7.35	69 (84.1%)	27.73 ± 3.45	EWGSOP2 2019	8/A
Salhöfer et al. (38)	Germany	2025	Retrospective CS	79	70 ± 14	64 (81%)	26.5 (24–30.8)	Sarcopenia index	6/B

CSS, cross-section study; CS, cohort study; BMI, body mass index; SMI, skeletal muscle mass index; SARC-F, strength, assistance with the walking, rising from a chair, climbing stairs, and falls questionnaire; EWGSOP2, European Working Group on Sarcopenia in Older People 2; ESMA, erector spinae muscle area; AWGS 2019, Asian Working Group on Sarcopenia in 2019; T4MI, the muscle index at T4; PMcas, cross-sectional area of the pectoralis muscles; PMI, pectoralis muscle index.

**TABLE 2:** Criteria and cut-off points to diagnose sarcopenia in individuals with IPF in the different studies.

Criteria	Main criteria definition	Lean muscle mass	Muscle function				
			Handgrip strength	Gait speed	SPPB	TUG	FTSST
EWGSOP2	Low muscle strength + low muscle mass; severity of sarcopenia involve low physical performance	ASMI: Male < 7.0 kg ⋅ m^–2^ Female < 5.5 kg ⋅ m^–2^	Male < 27 kg Female < 16 kg	≤ 0.8 m’s^–1^	≤ 8	≥ 20 s	
AWGS 2019	Low ASM + low muscle strength/low physical performance: severe sarcopenia: low ASM + low muscle strength + low physical performance	ASM: DXA (M: < 7.0 kg ⋅ m^–2^, Female < 5.4 kg ⋅ m^–2^) BIA (M: < 7.0 kg ⋅ m^–2^, Female < 5.7 kg.m^–2^)		< 1 m ⋅ s^–1^ (6MWT)	≤ 9		≥ 12 s
The lowest quartile of T4MI (Q4)	Patients’ T4Ml ranged from minimum to 25%		Male < 28 kg Female < 18 kg				
The lowest quartile of PMI (Q4)	Patients’ PMI ranged from minimum to 25%						
(The lowest quartile of SMI) Q4	Patients’ ASMI ranged from minimum to 25%						
(The lowest quartile of ESMA) Q4	Patients’ ESMA ranged from minimum to 25%						
SARC-F	≥ 4						
Pectoralis major cross-sectional area	Patients’ Pectoralis major cross-sectional are arranged from minimum to 25%						
(The lowest quartile of Sarcopenia index) Q4	Patients’ Sarcopenia index ranged from minimum to 25%						

SPPB, Short Physical Performance Battery; TUG, timed up and go test; FTSST, Five Times Sit-to-Stand Test; EWGSOP2, European Working Group on Sarcopenia in Older People 2019; SMI, skeletal muscle mass index; AWGS2019, Asian Working Group on Sarcopenia in 2019; ASM, appendicular skeletal muscle mass; DXA, dual-energy X-ray absorptiometry; BIA, bioelectrical impedance analysis; 6MWT, 6-min walk test; T4MI, the muscle index a tT4; PMcas, cross-sectional area of the pectoralis muscles; ESMA, erector spinae muscle area.

### 3.2 Literature quality assessment

The methodological quality of all included studies was evaluated based on the NOS and the JBI critical appraisal checklist (Table 1 and Supplementary Tables 4, 5). According to the respective appraisal standards, the overall quality of the studies was considered relatively high; seven were classified as grade A and eight as grade B.

### 3.3 Overall prevalence of sarcopenia in IPF patients

Figure 2 illustrates the estimated prevalence of sarcopenia among patients with IPF. The pooled prevalence derived from all included studies was calculated as 25% (95% CI: 23–28%). The I^2^ value was 44%, suggesting the absence of substantial heterogeneity across the studies. The funnel plot used to evaluate potential publication bias is presented in Supplementary Figure 1. Moreover, no evidence of publication bias was observed, as indicated by the symmetry of the funnel plot. This finding was further supported by Egger’s test (*p* = 0.787). Sensitivity analysis demonstrated that the exclusion of any individual study did not materially affect the pooled estimates, as shown in Supplementary Figure 2.

#### 3.3.1 Subgroup analysis

Available evidence suggests that factors such as literature quality, study design, ethnicity, age, and diagnostic criteria may influence the overall prevalence of sarcopenia. Based on subgroup analysis stratified by diagnostic criteria, the pooled prevalence of sarcopenia in IPF was 23% (95% CI: 0.17–0.32) when EWGSOP2 was applied, whereas the prevalence increased to 34% (95% CI: 0.28–0.42) when AWGS2019 was utilized as the diagnostic standard (Table 3 and Figure 2). Subgroup comparisons according to ethnicity indicated that the prevalence of sarcopenia among Caucasian IPF patients was slightly lower than that among their Asian counterparts (22% vs. 29%). Furthermore, when stratified by study design, retrospective studies yielded a pooled sarcopenia prevalence of 24% (95% CI: 0.21–0.28), while prospective studies reported a prevalence of 25% (95% CI: 0.19–0.33) (Table 3). Additional subgroup results based on age and literature quality are also summarized in Table 3.

**TABLE 3:** Subgroup analyses of sarcopenia in different conditions.

Subgroup analysis of sarcopenia (*n*)	Proportion (95% CI)	*P*-value	*I*^2^ (%)	*P*_*h*_
Overall (14)	0.25 (0.23, 0.28)	< 0.001	44%	0.04
**Diagnostic criteria**				
EWGSOP2 (4)	0.23 (0.17, 0.32)	< 0.001	55%	0.08
Others (7)	0.23 (0.20, 0.27)	< 0.001	0%	0.51
AWGS 2019 (3)	0.34 (0.28, 0.42)	< 0.001	12%	0.32
**Ethnicity**				
Caucasian (8)	0.22 (0.19, 0.26)	< 0.001	36%	0.14
Asian (6)	0.29 (0.25, 0.33)	< 0.001	24%	0.25
**Age**				
> 65 years (1)	NA	NA	NA	NA
< 65 years (13)	0.24 (0.21, 0.28)	< 0.001	39%	< 0.07
**Study quantity**				
High-quality/A (7)	0.23 (0.19, 0.26)	< 0.001	45%	0.09
Moderate-quantity/B (7)	0.27 (0.24, 0.31)	< 0.001	36%	0.16
**Design**				
Perspective (8)	0.25 (0.19, 0.33)	< 0.001	69%	< 0.01
Retrospective (6)	0.24 (0.22, 0.29)	< 0.001	0%	0.98

NA, not applicable; EWGSOP, The European Working Group on Sarcopenia in Older People; AWGS 2019, Asia Working Group for Sarcopenia; *P*_*h*_, *P*_*heterogeneity*_.

### 3.4 Sarcopenia assessment indicators in IPF patients

Five studies provided data on ESMCSA in patients with IPF. Upon pooling, the mean ESMCSA was calculated as 30.28 (95% CI: 26.57–34.00). HGS data were reported in six studies, yielding a pooled mean of 29.46 (95% CI: 26.00–32.92). Two studies contributed data on PhA, with a combined mean of 4.84 (95% CI: 4.72–4.96). For SMI, five studies were included, and the pooled mean value was 6.67 (95% CI: 6.30–7.04). TUG values were reported in two studies, producing a combined mean of 7.84 (95% CI: 7.07–8.61). Additionally, three studies reported PMCSA in IPF patients, with the aggregated mean calculated as 14.06 (95% CI: 10.22–17.90), as illustrated in Figure 3.

### 3.5 Impact of sarcopenia on pulmonary function, exercise capacity, and clinical outcomes in IPF patients

#### 3.5.1 Pulmonary function

Meta-analysis results demonstrated that individuals with IPF accompanied by sarcopenia exhibited markedly diminished pulmonary function metrics versus those with IPF alone (FVC: MD = −0.41L, 95% CI: −0.55 to 0.26; I^2^ = 8%, *P* < 0.0001, 6 studies; FEV1%: MD = −7.22%, 95% CI: −11.47 to −2.97; I^2^ = 37%, *P* = 0.0009, 5 studies; FEV1: MD = −0.26L, 95% CI: −0.39 to −0.13; I^2^ = 48%, *P* < 0.0001, 4 studies; FVC%: MD = −6.28%, 95% CI: −11.58 to −0.98; I^2^ = 67%, *P* = 0.02, 8 studies; DLCO%: MD = −7.81%, 95% CI: −11.94 to −3.69; I^2^ = 48%, *P* = 0.0002, 7 studies) (Figures 4A–E). It is worth noting that no statistically significant variation was detected in FEV1/FVC between sarcopenic IPF patients and those without sarcopenia: MD = 0.58%, 95% CI: −1.14 to 2.31; I^2^ = 21%, *P* = 0.51, 5 studies (Figure 4F).

#### 3.5.2 Exercise capacity

Data from five studies contributed to the analysis of 6MWT, revealing that individuals diagnosed with IPF and concomitant sarcopenia demonstrated markedly lower exercise tolerance versus those affected solely by IPF (6MWT: MD = −54.27 m, 95% CI: −91.31 to −17.24; I^2^ = 72%, *P* = 0.004, 5 studies) (Figure 4G). There was substantial heterogeneity for this outcome (I^2^ = 72%), and a random-effects model was applied. However, due to the limited sample size of the included literature, it is difficult to explore the source of heterogeneity through quantitative methods, such as meta-regression. We found that the pooled effects did not change after excluding a single study each time, which indicated that the results were stable.

#### 3.5.3 Clinical outcomes (descriptive analysis)

According to the findings of Salhöfer et al. (38), within the adjusted COX regression framework, significant and independent associations with overall survival were identified exclusively for the Fat index and Myosteatosis index, whereas the sarcopenia index did not emerge as a mortality predictor in individuals with IPF. Evidence from Sridhar et al. (16) indicated that among those diagnosed with IPF, the presence of concurrent sarcopenia was linked to a markedly reduced median survival duration compared to their counterparts without sarcopenia (19 vs. 61 months). Cabrera-César et al. (28) reported that in the context of IPF, the occurrence of sarcopenia led to a 4.89-fold elevation in the risk of adverse clinical outcomes within 12 months (hazard ratio [HR] = 4.89, 95% CI: 1.43–16.7, *P* = 0.011), with a 12-month survival rate of 100% in patients without sarcopenia, in contrast to 83% in those with the condition. In a 2-year observational cohort conducted by Çinkooğlu et al. (31), univariate COX regression analysis for all-cause mortality revealed that diminished HR was not associated with increased mortality risk (HR = 1.492, 95% CI: 1.002–2.222, *P* = 0.490). Furthermore, Fujikawa et al. (32) identified sarcopenia as a notable prognostic factor for all-cause mortality in a multivariable COX regression model after adjustments for sex, age, and FVC% (HR = 2.61, 95% CI: 1.51–4.51, *P* < 0.010).

### 3.6 Comparison of anti-fibrotic treatment numbers between sarcopenia and non-sarcopenia patients

**FIGURE 5:**
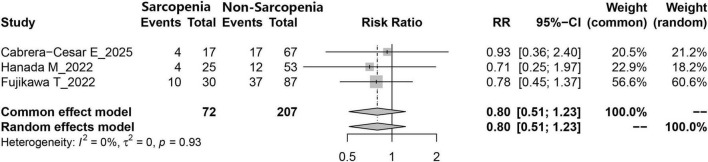
Comparison of the number of IPF patients receiving antifibrotic therapy (nintedanib or pirfenidone) between those with and without sarcopenia.

Data on the number of individuals with and without sarcopenia who received anti-fibrotic therapies involving nintedanib or pirfenidone were reported in three studies. According to the meta-analysis, no statistically significant disparity was observed in the administration patterns of respiratory treatment agents (nintedanib and pirfenidone) between the two groups (RR = 0.80, 95% CI: 0.51–1.23, *P* = 0.308) (Figure 5).

## 4 Discussion

This study systematically illustrated the prevalence of sarcopenia and its clinical implications among individuals diagnosed with IPF. By employing the EWGSOP2 and AWGS2019 diagnostic standards, alongside a comprehensive evaluation of clinicopathological parameters, sarcopenia was identified as not only commonly occurring in IPF patients but also strongly correlated with elevated disease severity and worsened clinical outcomes. Importantly, notable disparities in both exercise performance and pulmonary function were detected between those with and without sarcopenia, as determined by 6-min walk distance and pulmonary function assessments. These results emphasize the necessity of early detection and intervention for sarcopenia to enhance quality of life (QOL) and long-term outcomes in patients affected by IPF.

Although sarcopenia has been acknowledged as a complication associated with chronic illnesses such as chronic obstructive pulmonary disease, its occurrence and implications in patients with IPF remain insufficiently characterized. In this study, the detection rate of sarcopenia was identified as 25%, aligning closely with the previously reported prevalence of 26% (22). However, Hanada et al. (35). employed the AWGS 2019 diagnostic framework in a cohort of 78 Asian ILD patients and recorded a prevalence rate of 32%, exceeding that observed in the present analysis (25%). These discrepancies may stem from ethnic variation among study cohorts, distinct underlying pulmonary disorders, and inconsistencies in diagnostic criteria applied. It is noteworthy that in the studies incorporated in this analysis, the majority of participants were male. Prior investigations (12, 31) have conducted sex-specific analyses, given the known differences in muscle strength and mass between males and females. However, due to limited data and sex-specific diagnostic thresholds, subgroup analysis based on gender could not be carried out in the current study. The elevated incidence of sarcopenia in IPF patients may reflect a physiological response to fasting, malnutrition, or inactivity in the elderly population. Notably, malnutrition has been documented in approximately one-third of individuals with IPF (39). When compared to age-matched healthy controls, patients with IPF have exhibited markedly diminished physical activity levels (40). Moreover, common comorbid conditions observed in IPF, such as diabetes, renal failure, and heart failure, are also recognized contributors to sarcopenia (41). Therefore, systemic muscle degradation is more likely to develop in individuals affected by IPF.

Drug intervention is a significant factor contributing to the decline in nutritional status and alterations in body composition among patients with IPF. In this study, no statistically significant difference was observed in the distribution of antifibrotic agents between sarcopenic and non-sarcopenic individuals (RR = 0.80, *p* = 0.308), which is consistent with previous findings, indicating that the choice of antifibrotic treatment may not have a direct impact on the incidence of sarcopenia (33). It is noteworthy that nintedanib, a widely approved medication for progressive pulmonary fibrosis, has been repeatedly associated with appetite suppression in prior studies (33, 42). Therefore, its potential influence on sarcopenia development in IPF patients warrants careful consideration. Additionally, corticosteroids are frequently administered in the management of IPF, and their use has been linked to both sarcopenia and diminished exercise capacity (43). The administration of corticosteroids in the current analysis may act as a potential confounding factor. However, among the studies included, only Hanada et al. (35) reported corticosteroid usage stratified by sarcopenia status, revealing no significant difference between the two cohorts. In future meta-analyses, a greater number of primary studies should be incorporated to control for corticosteroid exposure and thereby minimize bias in the synthesized results.

This study revealed that sarcopenia-related assessment parameters, including ESMCSA, HGS, SMI, and PMCSA, were consistently lower in individuals diagnosed with IPF, indicating a heightened susceptibility to sarcopenia in this population. It may be inferred that reduced SMI is potentially associated with lower HRQOL and physical performance scores in IPF, which could adversely affect prognosis. In the investigation conducted by Ebihara et al. (36), significant correlations were observed between SMI, PMCSA, and physical capacity among IPF patients, highlighting the central role of physical performance in sarcopenia. These findings suggest that SMI and PMCSA may offer greater diagnostic utility than ESMCSA in evaluating sarcopenia in the context of IPF. PhA, derived via bioelectrical impedance analysis, serves as an additional key parameter for assessing body composition and is considered a sensitive biomarker of both health and nutritional status. Numerous studies have indicated that lower PhA values reflect diminished cellular integrity, reduced cell quantity, impaired membrane function, and are further associated with malnutrition, increased inflammatory and OS markers, decreased QOL, and unfavorable prognosis (44–46). Among individuals with IPF, those exhibiting reduced PhA were found to have lower values for FVC%, FEV1%, total lung capacity%, 6MWD, and SF-36 physical health subscale scores, as well as reduced fat-free mass index (39, 47). The study by Fernández-Jiménez et al. (48) reported that a PhA < 4.5° was associated with a 6.35-fold increase in mortality risk during a 12-month follow-up. Machado et al. (47) further substantiated that lower PhA levels in IPF were correlated with poorer pulmonary function (FVC, FEV1), reduced QOL, and an independent association with 6MWD. Nevertheless, in this study, the mean PhA among IPF patients was reported as 4.84°, falling within the typical reference range (4°–6°), possibly due to limitations in sample size. Further interventional research is warranted to clarify these observations.

This study additionally observed that both FVC and FEV1% values were markedly lower in individuals with sarcopenia compared to those without the condition. Such pulmonary function impairment aligns with previous findings (47, 49), reinforcing the considerable influence of muscle wasting on respiratory performance. The reduction in muscle mass and strength contributes to compromised lung function and the development of dyspnea. Conversely, diminished pulmonary capacity and dyspneic manifestations may restrict physical activity, potentially triggering muscular atrophic processes (50), thereby creating a self-perpetuating cycle. Therefore, this study identified a shorter 6MWD in sarcopenia patients, a metric widely employed for the objective evaluation of functional exercise capacity in individuals with moderate to severe pulmonary disorders (51). Nevertheless, there was substantial heterogeneity for this outcome (I^2^ = 72%). The potential heterogeneity may arise due to internal factors (such as IPF severity, study population, statistical methods, etc.) within each included study. Moreover, a greater reduction in DLCO% was also recorded among sarcopenia patients. In those diagnosed with IPF, decreased pulmonary diffusion capacity exerts a pronounced effect on exercise tolerance, while the contribution of declining muscle mass and function to this limitation should not be underestimated.

As the most prevalent subtype of ILD, IPF is supported by a broader body of evidence concerning nutritional factors and body composition dynamics. Prior studies have indicated that reduced muscle mass in patients with IPF is correlated with elevated mortality risk (10, 12, 48). However, in a cohort investigation by Sridhar et al. (16), a sarcopenia prevalence of 12.8% was reported, with no significant association observed between sarcopenia and mortality risk, possibly due to limitations in sample size and follow-up duration. Despite the well-established adverse implications of muscle wasting and its considerable prevalence among those with IPF, this condition remains frequently underdiagnosed. Such underrecognition may hinder the development and application of focused therapeutic interventions. The present findings underscore the necessity for systematic screening of sarcopenia in all patients with IPF. Successful implementation of this approach necessitates coordinated efforts among specialists in endocrinology, clinical nutrition, and pulmonary medicine, with the potential to enhance clinical outcomes and favorably influence disease trajectory.

Pulmonary rehabilitation is regarded as an effective therapeutic approach, with existing evidence supporting its capacity to enhance FVC, increase exercise tolerance, and improve QOL, while alleviating dyspnea in individuals with ILD (52, 53). Furthermore, properly tailored nutritional intervention is considered a key strategy for preserving skeletal muscle mass and myofiber integrity. In the context of chronic obstructive pulmonary disease, nutritional support has been demonstrated to increase lean body mass, enhance 6MWD, and improve both pulmonary function and QOL (54–56). Investigations focused on IPF populations have revealed that supplementation with vitamins D, C, and E can promote respiratory function and mitigate both inflammatory responses and overall survival (57). The integration of pulmonary rehabilitation with nutritional support may thus serve as a preferred clinical model to maintain optimal nutritional balance and prevent the onset of dynapenia or sarcopenia. However, due to the absence of relevant data in the included studies regarding changes in pulmonary function or prognosis following sarcopenia-specific interventions in IPF, no quantitative evaluation could be conducted to assess the impact of sarcopenia amelioration on survival quality and pulmonary function outcomes. Additionally, the findings of this study suggest that for IPF patients receiving interventions such as antifibrotic therapy, pulmonary rehabilitation, or nutritional support, clinicians should promptly document changes in the patients’ muscle mass and muscle strength.

Compared with Li et al. (22), the novelty of this meta-analysis includes the following four aspects. First, our study not only assessed the prevalence of sarcopenia in IPF but also elucidated its impact on pulmonary function, exercise capacity, and clinical outcomes in patients with IPF. The range of evaluated outcome measures is broader, providing clinicians with objective indicators to assess disease severity and prognostic outcomes in patients with IPF. Second, this meta-analysis included 15 studies, comprising 8 prospective and 7 retrospective studies. Both the number and quality of these studies surpass those of previous meta-analyses, making the findings more robust and applicable. Third, owing to the larger sample size, we were able to perform more comprehensive subgroup analyses. Finally, to mitigate publication bias, we utilized the complete data from all relevant studies, irrespective of their publication status. This approach is supported by Ferro et al. (58), who compared meta-analysis estimates derived solely from published reports with those obtained from pooled individual participant data. Their findings indicated that estimates based exclusively on published data tend to yield less precise summaries, likely as a result of publication bias. No significant publication bias was detected in the present study, indicating that the results are reliable.

Several limitations were recognized within the included studies. First, the included studies used highly heterogeneous diagnostic criteria for sarcopenia (EWGSOP2, AWGS 2019, CT-based indices, SARC-F questionnaire, etc.). While subgroup analyses were performed, the clinical comparability of the pooled prevalence remains questionable. The future research needs to unify the diagnostic criteria for sarcopenia to homogenize the observed population. Second, age-stratified data were not reported, although the prevalence of both IPF and sarcopenia is known to increase with age (59, 60), and older individuals, particularly those who are retired, often experience reduced physical activity, potentially introducing bias into the reported results. Similarly, the choice to pool both Asian and Caucasian cohorts, despite recognized ethnic differences in muscle mass reference values, might be a source of heterogeneity. Because of the small number of patients included in our study, no more stratified analyses or advanced analyses could be performed according to ethnicity. Third, individuals with severe health conditions who were unable to undergo pulmonary function testing or muscle mass evaluation were excluded, which may have led to selection bias. Additionally, prevalence figures may be influenced by environmental contexts—detection rates tend to be substantially higher in hospitalized patients and those residing in nursing homes or long-term care facilities compared to community-dwelling populations (61). These factors collectively may contribute to the underestimation of sarcopenia prevalence among patients with IPF. Four, chronic comorbidities are considered predisposing factors for sarcopenia, and substantial intergroup differences (between IPF patients with and without sarcopenia) in the prevalence of diabetes, hypertension, malignancies, and cardiac conditions could exist. These differences may be attributable to the lack of comprehensive comorbidity data in each study due to variations in the populations assessed. Five, the antifibrotics (appetite suppression by nintedanib) and corticosteroids (muscle wasting) may possess potential effects on sarcopenia. The antifibrotics (appetite suppression by nintedanib) and corticosteroids (muscle wasting) may possess potential effects on sarcopenia, but only one included study reported corticosteroid use stratified by sarcopenia status. Prospective studies are required to address this limitation. Moreover, this review prioritized the overinclusion of studies for a comprehensive picture of the existing evidence regarding sarcopenia in patients with IPF. As a result, the analysis included low quality studies, and this affected the interpretation of the findings. Additionally, most quantitative studies were cross-sectional, making it difficult to determine temporal directions of associations, which is necessary to establish causality. In future clinical trials, multiple measures can be improved to enhance research quality: (1) In terms of clinical outcome assessment, although the average survival period for IPF is 3–5 years, clinicians should document the short-term impact of sarcopenia on survival at the patient’s initial visit and conduct long-term evaluations of survival. Additionally, long-term follow-up observations should be made on the prognosis of patients undergoing antifibrotic medication. (2) For randomized controlled trials, researchers should provide detailed documentation of the random sequence generation process, allocation concealment, and implementation of blinding. Based on the study objectives and design, a scientifically justified sample size estimation should be performed. All these steps will improve the quality of publications and enhance their credibility.

## 5 Conclusion

This study indicates a notably high prevalence of sarcopenia among individuals with IPF and supports the necessity of early evaluation of muscle mass and strength within this patient population. Moreover, the presence of sarcopenia in IPF has been associated with impaired pulmonary function, reduced exercise performance, and less favorable prognostic outcomes. Looking ahead, interventions targeting sarcopenia may contribute to improved prognoses in patients diagnosed with IPF. Therefore, standardized sarcopenia diagnostic criteria and protocols are urgently needed to ensure accurate meta-analysis results and research conclusions. A prospective, multicenter prospective study with inclusion of sex-specific and comorbidity-adjusted analyses should be awaited in the future.

## Data Availability

The original contributions presented in this study are included in this article/Supplementary material, further inquiries can be directed to the corresponding authors.

## Supplementary material

Supplementary Figure 1 Funnel plot of sarcopenia incidence in IPF patients.

Supplementary Figure 2 Sensitivity analysis plot of sarcopenia incidence in IPF patients.
