# Supplementary material for: Sarcopenia in idiopathic pulmonary fibrosis: an updated systematic review and meta-analysis
Source: Front Med (Lausanne). 2025 Nov 4;12:1681237. doi: 10.3389/fmed.2025.1681237 (PMC12623184; doi:10.3389/fmed.2025.1681237)
Supplement: Supplementary file 2 [file Supplementary_file_2.docx]

**Supplementary Table 2 Search strategies in each database---** **July 7th, 2025**

| Database | Search strategies | Results |
| --- | --- | --- |
| **PubMed** | #1 "sarcopenia"[MeSH Terms]---13,028  #2 "humans"[MeSH Terms] AND ("Sarcopenia"[Title/Abstract] OR "Sarcopenias"[Title/Abstract] OR "muscle atrophy"[Title/Abstract] OR ("Sarcopenic"[Title/Abstract] OR "muscle attenuation"[Title/Abstract] OR "muscle loss"[Title/Abstract]) OR "muscle depletion"[Title/Abstract])---37,044  #3 #1 OR #2---37,842  #4 Idiopathic Pulmonary Fibrosis[MeSH Terms]---9,423  #5 Idiopathic Pulmonary Fibroses[Title/Abstract] OR Pulmonary Fibroses, Idiopathic[Title/Abstract] OR Idiopathic Fibrosing Alveolitis,Chronic Form[Title/Abstract] OR Fibrosing Alveolitis, Cryptogenic[Title/Abstract] OR Fibrocystic Pulmonary Dysplasia[Title/Abstract] OR Dysplasia, Fibrocystic Pulmonary[Title/Abstract] OR Fibrocystic Pulmonary Dysplasias[Title/Abstract] OR Pulmonary Dysplasia, Fibrocystic[Title/Abstract] OR Cryptogenic Fibrosing Alveolitis[Title/Abstract] OR Cryptogenic Fibrosing Alveolitides[Title/Abstract] OR Fibrosing Alveolitides, Cryptogenic[Title/Abstract] OR Pulmonary Fibrosis, Idiopathic[Title/Abstract] OR Familial Idiopathic Pulmonary Fibrosis[Title/Abstract] OR Idiopathic Pulmonary Fibrosis, Familial[Title/Abstract] OR Usual Interstitial Pneumonia[Title/Abstract] OR Usual Interstitial Pneumonias[Title/Abstract] OR Interstitial Pneumonitis, Usual[Title/Abstract] OR Pneumonitides, Usual Interstitial[Title/Abstract] OR Pneumonitis, Usual Interstitial[Title/Abstract] OR Usual Interstitial Pneumonitides[Title/Abstract] OR Usual Interstitial Pneumonitis[Title/Abstract]---17,528  #6 #4 OR #5---18,935  #7 #3 AND #6---35 | **35** |
| **Web of Science** | #1 (TS=(Sarcopenia)) OR AB=(Sarcopenia OR Sarcopenias OR Muscle atrophy OR Sarcopenic OR Muscle attenuation OR Muscle loss OR Muscle depletion)---69,806  #2 (TS=(Idiopathic Pulmonary Fibrosis)) OR AB=(Idiopathic Pulmonary Fibroses OR Pulmonary Fibroses, Idiopathic OR Idiopathic Fibrosing Alveolitis,Chronic Form OR Fibrosing Alveolitis, Cryptogenic OR Fibrocystic Pulmonary Dysplasia OR Dysplasia, Fibrocystic Pulmonary OR Fibrocystic Pulmonary Dysplasias OR Pulmonary Dysplasia, Fibrocystic OR Cryptogenic Fibrosing Alveolitis OR Cryptogenic Fibrosing Alveolitides OR Fibrosing Alveolitides, Cryptogenic OR Pulmonary Fibrosis, Idiopathic OR Familial Idiopathic Pulmonary Fibrosis OR Idiopathic Pulmonary Fibrosis, Familial OR Usual Interstitial Pneumonia OR Usual Interstitial Pneumonias OR Interstitial Pneumonitis, Usual OR Pneumonitides, Usual Interstitial OR Pneumonitis, Usual Interstitial OR Usual Interstitial Pneumonitides OR Usual Interstitial Pneumonitis)---18,759  #3 #1 AND #2---115 | **115** |
| **Embase** | #1 'sarcopenia'/exp OR sarcopenia:ti,ab,kw OR sarcopenias:ti,ab,kw OR 'muscle atrophy':ti,ab,kw OR sarcopenic:ti,ab,kw OR 'muscle attenuation':ti,ab,kw OR 'muscle loss':ti,ab,kw OR 'muscle depletion':ti,ab,kw ---59,173  #2 'idiopathic pulmonary fibrosis'/exp OR 'idiopathic pulmonary fibroses':ti,ab,kw OR 'pulmonary fibroses, idiopathic':ti,ab,kw OR 'idiopathic fibrosing alveolitis,chronic form':ti,ab,kw OR 'fibrosing alveolitis, cryptogenic':ti,ab,kw OR 'fibrocystic pulmonary dysplasia':ti,ab,kw OR 'dysplasia, fibrocystic pulmonary':ti,ab,kw OR 'fibrocystic pulmonary dysplasias':ti,ab,kw OR 'pulmonary dysplasia, fibrocystic':ti,ab,kw OR 'cryptogenic fibrosing alveolitis':ti,ab,kw OR 'cryptogenic fibrosing alveolitides':ti,ab,kw OR 'fibrosing alveolitides, cryptogenic':ti,ab,kw OR 'pulmonary fibrosis, idiopathic':ti,ab,kw OR 'familial idiopathic pulmonary fibrosis':ti,ab,kw OR 'idiopathic pulmonary fibrosis, familial':ti,ab,kw OR 'usual interstitial pneumonia':ti,ab,kw OR 'usual interstitial pneumonias':ti,ab,kw OR 'interstitial pneumonitis, usual':ti,ab,kw OR 'pneumonitides, usual interstitial':ti,ab,kw OR 'pneumonitis, usual interstitial':ti,ab,kw OR 'usual interstitial pneumonitides':ti,ab,kw OR 'usual interstitial pneumonitis':ti,ab,kw ---41,149  #3 #1 AND #2---106 | **106** |
| **Cochrane library** | #1 MeSH descriptor: [Sarcopenia] explode all trees---1,038  #2 (Sarcopenia OR Sarcopenias OR Muscle atrophy OR Sarcopenic OR Muscle attenuation OR Muscle loss OR Muscle depletion):ti,ab,kw---12,525  #3 #1 OR #2---12,525v  #4 MeSH descriptor: [Idiopathic Pulmonary Fibrosis] explode all trees---610  #5 (Idiopathic Pulmonary Fibroses OR Pulmonary Fibroses, Idiopathic OR Idiopathic Fibrosing Alveolitis,Chronic Form OR Fibrosing Alveolitis, Cryptogenic OR Fibrocystic Pulmonary Dysplasia OR Dysplasia, Fibrocystic Pulmonary OR Fibrocystic Pulmonary Dysplasias OR Pulmonary Dysplasia, Fibrocystic OR Cryptogenic Fibrosing Alveolitis OR Cryptogenic Fibrosing Alveolitides OR Fibrosing Alveolitides, Cryptogenic OR Pulmonary Fibrosis, Idiopathic OR Familial Idiopathic Pulmonary Fibrosis OR Idiopathic Pulmonary Fibrosis, Familial OR Usual Interstitial Pneumonia OR Usual Interstitial Pneumonias OR Interstitial Pneumonitis, Usual OR Pneumonitides, Usual Interstitial OR Pneumonitis, Usual Interstitial OR Usual Interstitial Pneumonitides OR Usual Interstitial Pneumonitis):ti,ab,kw---1,782  #6 #4 OR #5---1,794  #7 #3 AND #6---6 | **6** |
| **CNKI** | #1 SU=(Sarcopenia) OR TKA=(Sarcopenia OR Sarcopenias OR Muscle atrophy OR Sarcopenic OR Muscle attenuation OR Muscle loss OR Muscle depletion)---26,838  #2 SU=(Idiopathic Pulmonary Fibrosis) OR TKA=(Idiopathic Pulmonary Fibroses OR Pulmonary Fibroses, Idiopathic OR Idiopathic Fibrosing Alveolitis,Chronic Form OR Fibrosing Alveolitis, Cryptogenic OR Fibrocystic Pulmonary Dysplasia OR Dysplasia, Fibrocystic Pulmonary OR Fibrocystic Pulmonary Dysplasias OR Pulmonary Dysplasia, Fibrocystic OR Cryptogenic Fibrosing Alveolitis OR Cryptogenic Fibrosing Alveolitides OR Fibrosing Alveolitides, Cryptogenic OR Pulmonary Fibrosis, Idiopathic OR Familial Idiopathic Pulmonary Fibrosis OR Idiopathic Pulmonary Fibrosis, Familial OR Usual Interstitial Pneumonia OR Usual Interstitial Pneumonias OR Interstitial Pneumonitis, Usual OR Pneumonitides, Usual Interstitial OR Pneumonitis, Usual Interstitial OR Usual Interstitial Pneumonitides OR Usual Interstitial Pneumonitis) ---9482  #3 #1 AND #2---23 | **23** |
