# Supplementary material for: Sarcopenia in idiopathic pulmonary fibrosis: an updated systematic review and meta-analysis
Source: Front Med (Lausanne). 2025 Nov 4;12:1681237. doi: 10.3389/fmed.2025.1681237 (PMC12623184; doi:10.3389/fmed.2025.1681237)
Supplement: Supplementary file 3 [file Supplementary_file_3.docx]

**Supplementary Table 3 The checklist items for Joanna Briggs's critical appraisal tool for prevalence studies.**

|  | Yes | No | Unclear | Not applicable |
| --- | --- | --- | --- | --- |
| 1. Was the sample frame appropriate to address the target population? | □ | □ | □ | □ |
| 1. Were study participants sampled in an appropriate way? | □ | □ | □ | □ |
| 1. Was the sample size adequate? | □ | □ | □ | □ |
| 1. Were the study subjects and the setting described in detail? | □ | □ | □ | □ |
| 1. Was the data analysis conducted with sufficient coverage of the identified sample? | □ | □ | □ | □ |
| 1. Were valid methods used for the identification of the condition? | □ | □ | □ | □ |
| 1. Was the condition measured in a standard, reliable way for all participants? | □ | □ | □ | □ |
| 1. Was there appropriate statistical analysis? | □ | □ | □ | □ |
| 1. Was the response rate adequate, and if not, was the low response rate managed appropriately? | □ | □ | □ | □ |
