# Supplementary material for: Sarcopenia in idiopathic pulmonary fibrosis: an updated systematic review and meta-analysis
Source: Front Med (Lausanne). 2025 Nov 4;12:1681237. doi: 10.3389/fmed.2025.1681237 (PMC12623184; doi:10.3389/fmed.2025.1681237)
Supplement: Supplementary file 4 [file Supplementary_file_4.docx]

**Supplementary Table 4 Quality assessment(Joanna Briggs's critical appraisal tool) of the included studies.**

| **Author** | **Checklist item** | | | | | | | | | **Score**  **(Out of 9)** |
| --- | --- | --- | --- | --- | --- | --- | --- | --- | --- | --- |
|  | **1** | **2** | **3** | **4** | **5** | **6** | **7** | **8** | **9** |  |
| Nakano A | 1 | 0 | 0 | 0 | 1 | 1 | 1 | 1 | 1 | 6 |
| Moon SW | 1 | 0 | 1 | 1 | 1 | 1 | 1 | 1 | 1 | 8 |
| Ebihara K | 1 | 0 | 0 | 1 | 1 | 1 | 1 | 1 | 1 | 7 |
| Faverio P | 1 | 0 | 1 | 1 | 1 | 1 | 1 | 1 | 1 | 8 |
| Hanada M | 1 | 0 | 0 | 0 | 1 | 1 | 1 | 1 | 1 | 6 |
| Fujikawa T | 1 | 0 | 0 | 0 | 0 | 1 | 1 | 1 | 1 | 5 |
| Çinkooğlu A | 1 | 0 | 0 | 0 | 0 | 1 | 1 | 1 | 1 | 5 |
| Holst M | 1 | 0 | 0 | 1 | 1 | 1 | 1 | 1 | 1 | 7 |
| Fujita K | 1 | 0 | 0 | 0 | 1 | 1 | 1 | 1 | 1 | 6 |
| Ohkubo H | 1 | 0 | 0 | 0 | 0 | 1 | 1 | 1 | 1 | 5 |
| Sridhar M | 1 | 1 | 0 | 1 | 1 | 1 | 1 | 1 | 1 | 8 |
| Cabrera-César E | 1 | 0 | 0 | 0 | 1 | 1 | 1 | 1 | 1 | 6 |
| Ibarra-Fernández AA | 1 | 1 | 0 | 1 | 1 | 1 | 1 | 1 | 1 | 8 |
| Sanmartín-Sánchez A | 1 | 1 | 0 | 0 | 1 | 1 | 1 | 1 | 1 | 7 |
| Salhöfer L | 1 | 0 | 0 | 0 | 1 | 1 | 1 | 1 | 1 | 6 |
