# Supplementary material for: Sarcopenia in idiopathic pulmonary fibrosis: an updated systematic review and meta-analysis
Source: Front Med (Lausanne). 2025 Nov 4;12:1681237. doi: 10.3389/fmed.2025.1681237 (PMC12623184; doi:10.3389/fmed.2025.1681237)
Supplement: Supplementary file 5 [file Supplementary_file_5.docx]

**Supplementary Table 5 Risk of bias of the included studies using the Newcastle-Ottawa Scale.**

| First author | Selection | | | | Comparability | Outcome | | |
| --- | --- | --- | --- | --- | --- | --- | --- | --- |
|  | Q1 | Q2 | Q3 | Q4 | Q1 | Q1 | Q2 | Q3 |
| Nakano A | * | * |  | * | * | * | * |  |
| Moon SW | * | * | * | * | ** | * | * |  |
| Ebihara K | * | * | * | * | * | * | * |  |
| Faverio P | * | * | * | * | ** | * | * | * |
| Hanada M | * |  |  | * | * | * | * |  |
| Fujikawa T | * | * |  | * | * | * | * |  |
| Çinkooğlu A | * | * |  | * | * | * | * |  |
| Holst M | * | * | * | * | * | * | * |  |
| Fujita K | * | * |  | * | * | * | * |  |
| Ohkubo H | * | * |  | * | * | * | * |  |
| Sridhar M | * | * | * | * | ** | * | * |  |
| Cabrera-César E | * | * |  | * | * | * | * |  |
| Ibarra-Fernández AA | * | * | * | * | ** | * | * |  |
| Sanmartín-Sánchez A | * | * | * | * | ** | * | * |  |
| Salhöfer L | * | * |  | * | * | * | * |  |

NEWCASTLE - OTTAWA QUALITY ASSESSMENT SCALE

CASE CONTROL STUDIES

Note: A study can be awarded a maximum of one star for each numbered item within the Selection and Exposure categories. A maximum of two stars can be given for Comparability.

Selection

1) Is the case definition adequate?

a) yes, with independent validation 

b) yes, eg record linkage or based on self reports

c) no description

2) Representativeness of the cases

a) consecutive or obviously representative series of cases 

b) potential for selection biases or not stated

3) Selection of Controls

a) community controls 

b) hospital controls

c) no description

4) Definition of Controls

a) no history of disease (endpoint) 

b) no description of source

Comparability

1) Comparability of cases and controls on the basis of the design or analysis

a) study controls for ______________ (Select the most important factor.) 

b) study controls for any additional factor. (This criteria could be modified to indicate specific control for a second important factor.)

Exposure

1) Ascertainment of exposure

a) secure record (eg surgical records) 

b) structured interview where blind to case/control status 

c) interview not blinded to case/control status

d) written self report or medical record only

e) no description

2) Same method of ascertainment for cases and controls

a) yes 

b) no

3) Non-Response rate

a) same rate for both groups 

b) non respondents described

c) rate different and no designation
