# Supplementary figures and images for: Sarcopenia in idiopathic pulmonary fibrosis: an updated systematic review and meta-analysis
Source: Front Med (Lausanne). 2025 Nov 4;12:1681237. doi: 10.3389/fmed.2025.1681237 (PMC12623184; doi:10.3389/fmed.2025.1681237)

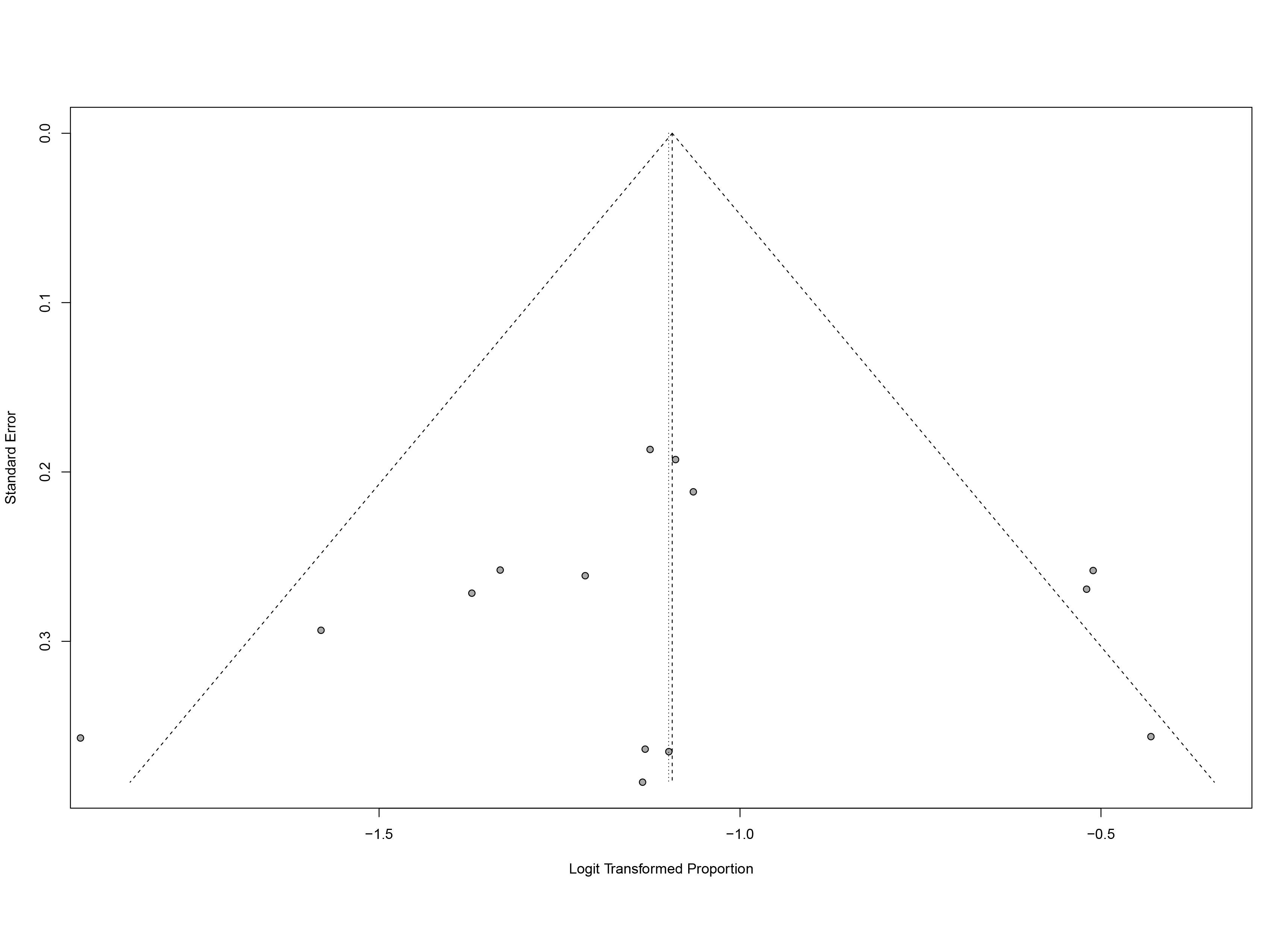

Supplement: Supplementary Figure 1 — Funnel plot of sarcopenia incidence in IPF patients. [file Image_1.tif]

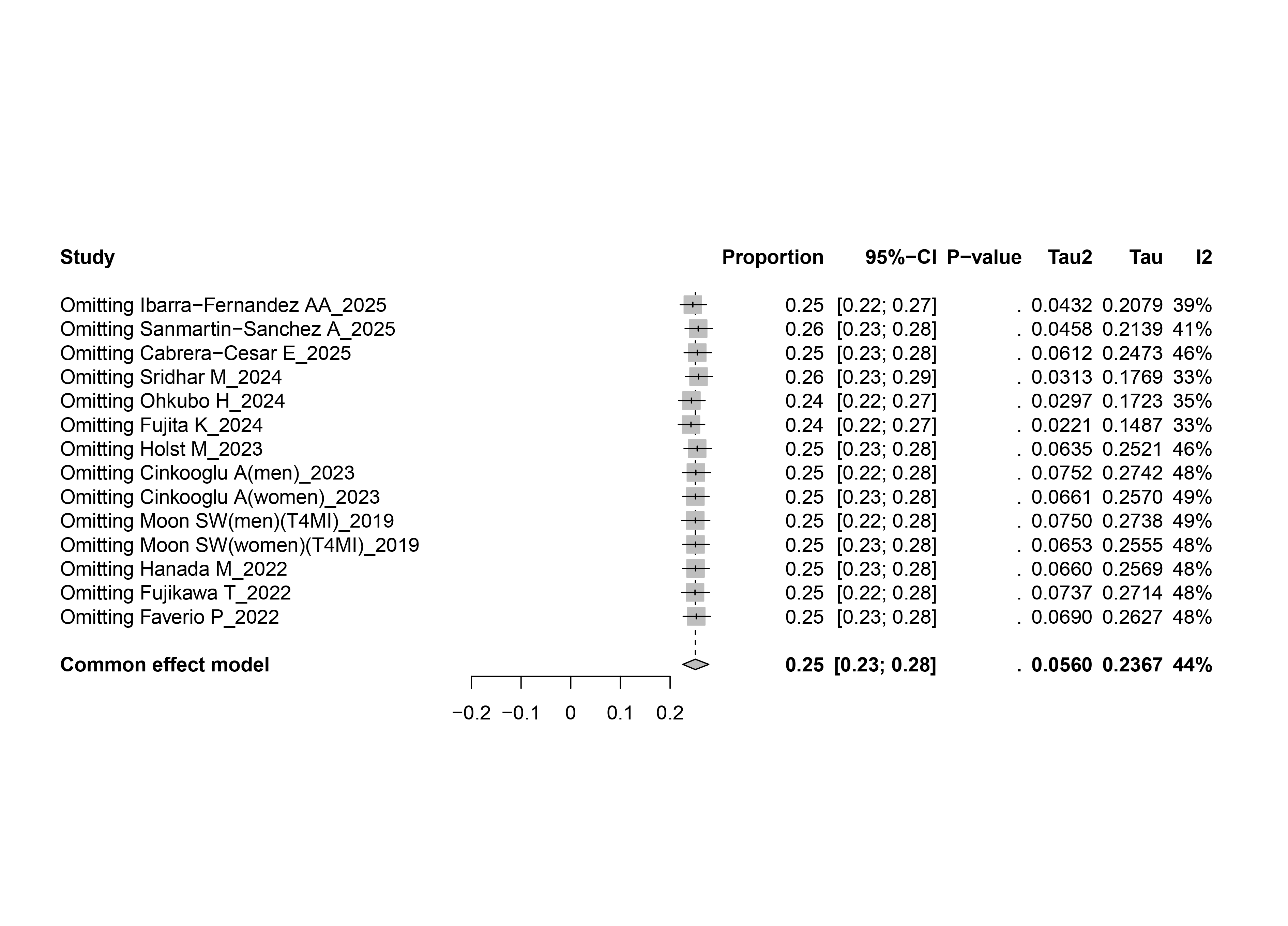

Supplement: Supplementary Figure 2 — Sensitivity analysis plot of sarcopenia incidence in IPF patients. [file Image_2.tif]
